# Women’s experiences of seeking healthcare for abdominal pain in Ireland: a qualitative study

**DOI:** 10.1186/s12905-024-02995-3

**Published:** 2024-03-07

**Authors:** Eibhlín B. Windrim, Brian E. McGuire, Hannah Durand

**Affiliations:** 1 School of Psychology, University of Galway, Galway, Ireland; 2 Centre for Pain Research, University of Galway, Galway, Ireland; 3 Division of Psychology, University of Stirling, Stirling, Scotland

**Keywords:** Abdominal pain, Women’s health, Health inequities, Health care seeking behaviour, Health services accessibility, Qualitative research

## Abstract

**Background:**

Evidence suggests that women’s abdominal pain is more likely to be minimised or dismissed by healthcare professionals than men’s. This can have a detrimental impact on health-related outcomes as well as quality of life. The aim of this study was to explore women’s experiences of seeking healthcare for abdominal pain in Ireland.

**Method:**

A qualitative design and opportunity sampling approach were employed in this study. Fourteen women living in Ireland with experience of seeking healthcare for abdominal pain took part in one-to-one semi-structured interviews via video-conferencing software. Data were analysed using reflexive thematic analysis.

**Results:**

Four themes were constructed from the data: [1] “Just Get on with It” – Normalisation and Invalidation; [2] “Bad Enough”? Costs of (Not) Seeking Help; [3] “Fight Your Case,” Fight for Care; and [4] “Out of the Loop” – Systemic Barriers to Care. Perceived invalidation of pain by healthcare professionals was common, as was internalised normalisation of pain. This created challenges when negotiating pain management solutions. Despite functional interference, participants felt their pain needed to reach an extreme level of severity before seeking help. Costs of private healthcare were implicated in delayed help-seeking. Participants felt the onus was on them to fight for care. Social support and information-seeking facilitated participants in this fight while systemic issues were identified as barriers to adequate care. Despite their frustrations, participants expressed empathy for healthcare professionals operating in a flawed system.

**Conclusions:**

Participants described mostly negative experiences of seeking healthcare for abdominal pain, characterised by dismissal of symptoms and internalisation of normative views of women’s pain as less worthy of care. These experiences reinforced participants’ views that self-advocacy is essential to access care for their pain. There are systemic issues at play within the Irish healthcare system that limit women’s ability to access abdominal pain management support. Education and training for healthcare professionals on the Gender Pain Gap and its implications for patient care, as well as clear referral pathways for women presenting with abdominal pain, may help to ensure more equitable healthcare delivery for individuals with abdominal pain in Ireland.

**Supplementary Information:**

The online version contains supplementary material available at 10.1186/s12905-024-02995-3.

Abdominal pain refers to pain that occurs between the chest and the pelvic area. It can present as cramping, dull, aching, sharp, or stabbing pain that may be either constant or intermittent. Abdominal pain can be acute or chronic. Acute abdominal pain typically presents suddenly and may be associated with nausea or vomiting, and is often attributable to infection, inflammation, perforation, or obstruction [1]. Chronic abdominal pain lasts for greater than three months’ duration [2], and may be indicative of underlying pathology [3]; however, the underlying cause of abdominal pain cannot be specified in about one third of patients [4]. Abdominal pain often affects functional capacity and quality of life and is a leading cause of healthcare utilisation internationally [3].

Abdominal pain affects between 22% and 25% of the population, with a higher prevalence among women (24%) than men (17%) [5, 6]. Gastrointestinal issues such as irritable bowel syndrome (IBS) and inflammatory bowel disease (IBD) account for much of the abdominal pain experienced by both men and women, with sex-based differences in pathogenesis and presentation being well established [7, 8]. However, abdominal pain is also a common symptom of a wide variety of gynaecological conditions, such as endometriosis, fibroids, ovarian syndromes, and pelvic inflammatory disease [3, 9]. Pain in the abdomen during menstruation (i.e., dysmenorrhea) secondary to various gynaecological disorders and/or as a primary form of disease is also common and debilitating [10, 11].

## Healthcare and the gender pain gap

The Gender Pain Gap refers to the phenomenon in which women’s pain is more poorly understood and undertreated compared to pain in men due to systemic gaps and biases. Clinical trials and other types of health research have traditionally adopted a ‘male as default’ or andronormative approach [12], which limits our understanding of pain conditions that predominantly affect women or how certain conditions affect men and women differently. This phenomenon has also contributed to the normalisation of women’s pain and gender biases within healthcare [13], which may result in women not seeking help for debilitating symptoms and impact the nature and quality of healthcare interactions for those who do. In their systematic review on gender bias in healthcare, Samulowitz and colleagues [12] identified a distinct pattern of gendered norms in the chronic pain literature. Women were presented as having greater bodily awareness and therefore heightened sensitivity to pain relative to men. It was also commonly suggested that pain without an identifiable cause is a natural characteristic of the female body. Additionally, women’s pain is more likely to be considered hysterical or psychological in origin, and as such is more likely to be described as ‘medically unexplained.’

Sex and gender influence the presentation of pain, which in turn influences patient care. In general, pain is underestimated by healthcare professionals (HCPs); however, evidence suggests male patients’ pain is overestimated relative to female patients’ pain [14]. The ability of HCPs to accurately assess patient pain is often compromised by pre-existing gender biases, which determine how pain is addressed and treated in healthcare settings [15]. Gender variations in healthcare experiences have been observed for abdominal pain, specifically. In their study on gender disparity in the analgesic treatment of acute abdominal pain in emergency departments, Chen et al. [16] reported that, although women were more likely to present with abdominal pain than men, they were less likely to receive pain relief than men reporting similar pain scores. Women’s abdominal pain is often considered less serious than men’s, oftentimes being dismissed as ‘just’ dysmenorrhea [17] without due regard for severity or impact of the pain [18]. Due to the normalisation of dysmenorrhea, both socially and medically, perceived dismissal of women’s abdominal pain symptoms in healthcare contexts is common [16, 17, 19, 20]. Experiences of dismissal by HCPs can lead to feelings of guilt, inadequacy, and helplessness, which can impact self-efficacy and resilience as well as future help-seeking behaviour. In Ireland, women have been demonstrated to delay seeking necessary healthcare assistance for a month or more on average [21]. Delay was influenced by women’s knowledge, beliefs, and social factors; in particular, women were more likely to delay when they anticipated that they would not be heard by HCPs. Validation from HCPs is important to ensure patients feel comfortable in seeking help [22, 23]. Anticipated or experienced invalidation by HCPs may explain the greater tendency for women to self-advocate and utilise more self-advocacy strategies than men [24]. Women may develop these skills and strategies, particularly health information-seeking [25], in an attempt to overcome the challenges they face in accessing care.

The Gender Pain Gap is a systemic issue; therefore, it is useful to consider the various contextual determinants that may influence the experiences of women with pain within specific healthcare systems and distinct socio-political and cultural landscapes. Ireland has a two-tier healthcare system, consisting of both private and public healthcare services [26, 27]. This type of system can exacerbate healthcare disparities and result in fragmentation of care and strain on public service resources [27]. This two-tier system has its origins in the early 1900s and is in large part a consequence of the Catholic church’s historical alliance with the medical profession in Ireland [28]. Although modern Ireland may be described as socially liberal, some would argue its conservative history can still be seen in the church’s continued influence over Irish healthcare policy and delivery [28], which may disproportionately impact female patients. Repeated women’s healthcare-related scandals, both historical [29, 30] and contemporary [31, 32], have demonstrated Ireland’s poor track record in providing adequate care to women. Although there have been substantive changes to women’s health policy in response to public outcry in recent years, the extent to which these have impacted women’s healthcare experiences to date is unclear. Cumulatively these factors are likely to influence women’s pain-related help-seeking behaviour, as well as the quality of care they receive. However, there is a dearth of research on the experiences of women seeking healthcare for abdominal pain in the Irish context to date.

Cumulatively the literature suggests that there are clear gender-based disparities present in healthcare systems, which can negatively impact the experiences of women with pain. In particular, women who present with abdominal pain are likely to have their pain experiences dismissed or invalidated, and to have their conditions misdiagnosed and undertreated. However, there is a dearth of research on the experiences of women with abdominal pain accessing healthcare in the Irish context, specifically. Given the unique interplay of healthcare system-related factors, social and cultural factors, and recent changes in women’s health policies and practice [33, 34], understanding women’s experiences of seeking help for abdominal pain in Ireland can provide important insights to help optimise healthcare delivery nationally, while contributing to the broader global discourse on sex- and gender-based disparities in healthcare. The current study aimed to explore women’s experiences of accessing healthcare for abdominal pain in Ireland using a qualitative approach.

## Method

### Participants and recruitment

Women over the age of 18 years who were resident in Ireland and had attended Irish healthcare services for abdominal pain were invited to take part in the study. No restrictions were placed on the type, severity, or duration of abdominal pain. A convenience sampling approach was taken. Study information was disseminated via social media platforms (Reddit, LinkedIn, Facebook, and Twitter) and flyers were posted on the university campus and at health-related conferences. Snowball sampling was also used, whereby participants were encouraged to share the study information with others who may have been eligible to take part. Recruitment took place over a period of four months (March–June 2023).

A total of 14 women took part in the study. Data collection ceased once data adequacy (i.e., data sufficient to answer the research question in both amount and variety [35]) was achieved according to evaluation by all members of the research team. Participant information is presented in Table 1. Only age groups are reported to better protect participant anonymity.

**Table 1:** Participant characteristics

ID no.	Pseudonym	Age group	Nature of pain
01	Alice	18–29	Dysmenorrhea, PCOS, ruptured cysts
02	Barbara	18–29	Dysmenorrhea, unidentified gastrointestinal issues, IBS
03	Charlotte	18–29	Unidentified abdominal pain, dysmenorrhea
04	Danielle	18–29	Unidentified abdominal pain
05	Emily	18–29	Endometriosis, PCOS
06	Fiona	30–39	Interstitial cystitis, PCOS, IBS, fibroids, endometriosis, PMDD
07	Grace	18–29	Dysmenorrhea, unidentified abdominal pain, PMDD
08	Hilary	18–29	PCOS, endometriosis, adenomyosis
09	Isabelle	18–29	Unidentified gastrointestinal issue
10	Jessica	18–29	Unidentified abdominal pain
11	Katherine	30–39	Endometriosis, fibroids
12	Louise	50–59	Appendicitis, unidentified gastrointestinal issue
13	Marie	18–29	Dysmenorrhea, unidentified abdominal pain
14	Natalie	18–29	Unidentified gastrointestinal issue

*Note:* PCOS = Polycystic ovary syndrome; PMDD = premenstrual dysphoric disorder

### Study design and procedure

A qualitative interpretative approach to the research was employed. One-to-one interviews were used to explore participants’ experiences of the healthcare system in Ireland when presenting with abdominal pain. Interviews were conducted by the lead author (EBW) via video-conferencing platforms Zoom and Microsoft Teams, where participants were invited to remain on- or off-camera, as desired. Interviews were semi-structured and followed a previously prepared interview guide (see *Additional file 1*). Time was allocated at the start of each interview to inform the participant of the study’s purpose and content, to obtain informed consent, and to build rapport with the participant. Participants were encouraged to ask any questions they had concerning the research and their participation and were reminded that they were not obligated to share any information with which they were uncomfortable. The interviews lasted an average of 31 min (range: 15–55 min). Interviews were audio-recorded and transcribed verbatim by the lead author (EBW) for analysis. Transcripts were validated by a senior author (HD) to ensure they accurately captured participants’ verbal and non-verbal communication (e.g., pauses, laughter). After transcription, audio recordings were destroyed. Ethical approval was obtained from the School of Psychology Research Ethics Committee at the University of Galway (Ref: SREC-17-Feb-23). Ethical standards of the institutional research committee were upheld throughout the research process. Data was handled and stored in accordance with requirements set out by General Data Protection Regulation (GDPR).

### Data analysis

Reflexive thematic analysis according to Braun and Clarke [36–38] was used to analyse the data. An inductive approach was taken, whereby themes were entirely derived from the data. This analysis approach comprised the following six stages:

1. *Familiarising yourself with the data*: Interview recordings were transcribed verbatim by the lead author, facilitating familiarisation with the data.
2. *Generating initial codes*: A semantic approach was taken to generate codes, by extracting relevant phrases and sentences from the data, establishing recurrences throughout the data.
3. *Searching for themes*: Relationships between codes were considered using visual representations. Codes were then divided based on similarity, creating themes.
4. *Reviewing themes*: The coded data extracts were reviewed for each theme. The validity of each theme was reviewed in relation to the dataset and the research topic, to ensure the analysis provided an accurate representation of the data and addressed the research aim.
5. *Defining and naming themes*: Each theme was given an operational name and definition. Each theme was defined by identifying its context and depth in relation to the research question.
6. *Producing the report*: A detailed account of each theme supported with extracts from the transcripts was established within the final report.

The analysis was undertaken by the lead author, with support from a senior author (HD) with extensive experience in qualitative health research. The lead author transcribed the interviews, developed the codes, and generated an initial set of themes. The research team had frequent meetings to discuss the data and analysis throughout this phase. Codes, categories, and initial themes were reviewed by a senior author (HD) for comprehensiveness, coherence, and grounding in the data. Any proposed refinements to the themes were agreed among all authors.

The credibility of the findings was ensured through prolonged engagement with the data and frequent in-depth discussions among the research team during the data collection, analysis, and writing processes. Peer debriefing, whereby findings and interpretations were discussed with colleagues not directly involved in the research to help identify any potential biases, challenge assumptions, and gain additional relevant insights, was practiced during the analysis stage to enhance the credibility of the findings [39].

### Reflexivity

Reflexivity was practiced throughout the research process. Reflexivity involves researchers acknowledging and critically reflecting their role in shaping the research and its findings, including how personal beliefs, values, and experiences may impact data collection, analysis, and interpretation [40]. The lead author and interviewer (EBW) was a young cisgender female living in Ireland with experience of the Irish healthcare system. This may have facilitated rapport-building with participants, who largely shared similar characteristics. Reflexive writing was employed to record the researchers’ viewpoints and decisions throughout the research process, establishing a reference log for later stages of the research. This also enhanced the dependability and confirmability of the findings [41]. The research team also met frequently to practice collaborative reflexivity, questioning each other’s assumptions and decisions from their individual perspectives across all stages of the research. Peer debriefing, as described above, also facilitated reflexivity.

## Results

**Fig. 1:**
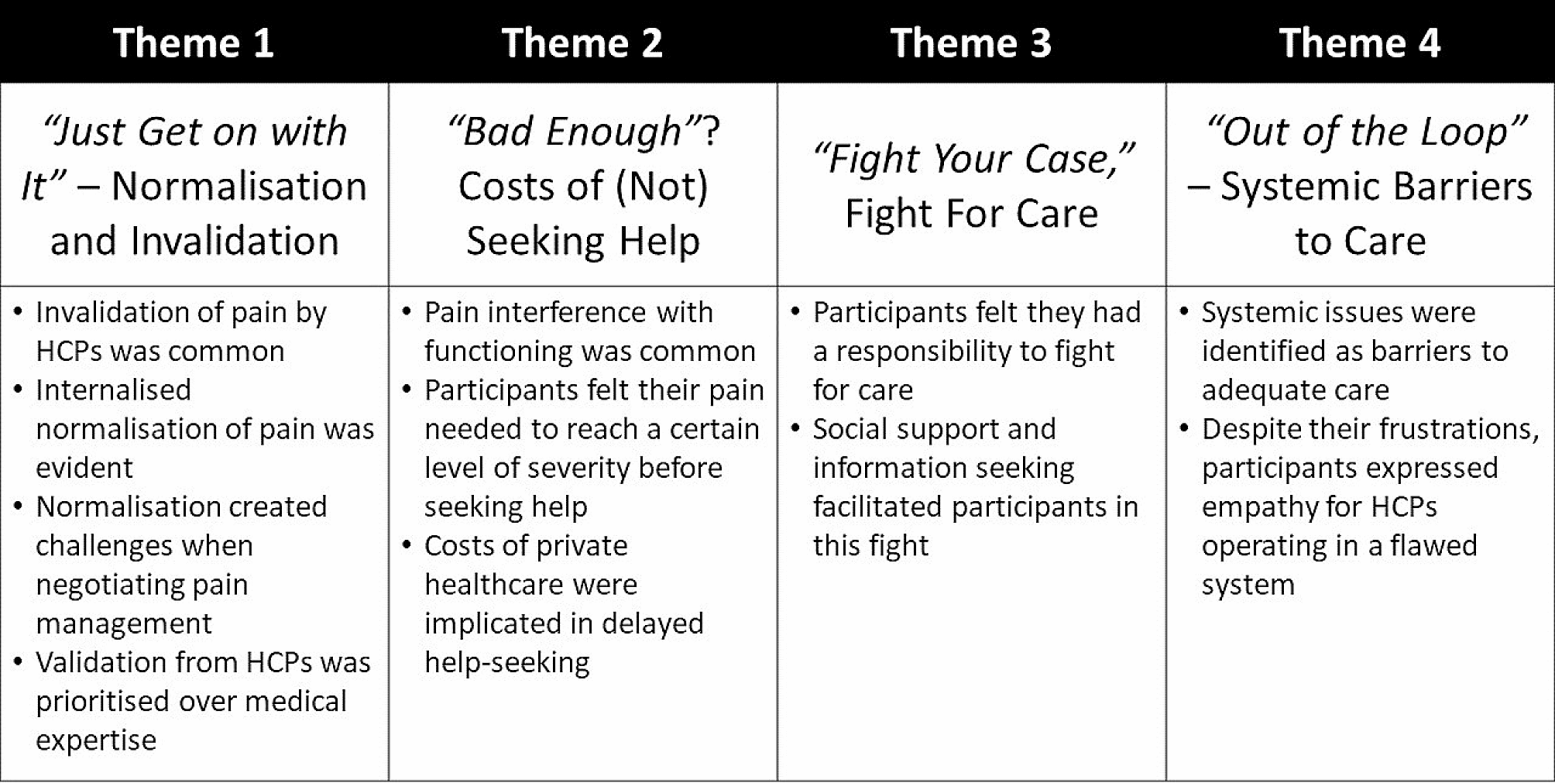
Summary of themes

Four themes were constructed from the data. Themes are outlined below (see Fig. 1) and illustrated using supporting quotations.

### Theme 1 – “Just Get on with It” – Normalisation and Invalidation

All participants described instances of not feeling listened to by HCPs in the past. For the majority, this was explicitly linked to dismissal and invalidation of their symptoms and the normalisation of abdominal pain in women. Participants felt that their doctors saw their pain as an inherent part of their womanhood and therefore not needing treatment, “like having a uterus can discredit other ailments,” (P07).

“‘Oh, it’s normal, and a lot of people have really painful periods, it’s just part of being a woman, blah blah blah…’” (P01, quoting HCP)

“‘Oh, you’ve a sore tummy? Welcome to womanhood.’” (P05, quoting HCP)

“I usually leave the doctor just feeling really confused and kind of, like, dismissed.” (P02)

Some participants internalised the normalisation of their pain, which influenced the ways in which they understood and described their experiences. These participants minimised their own symptoms during the interviews. Many regarded their pain as normal and described themselves as simply “unlucky” (P01) or as having a “low pain tolerance” (P01) relative to others. For most, this internalised normalisation of pain resulted from having their abdominal pain invalidated in their initial interactions with HCPs.

“I was like, ‘oh, it’ll pass.’” (P05)

“We might be feeling really bad, but you will try to minimise it yourself.” (P11)

The narrative that abdominal pain is something women need to just deal with was apparent in most of the interviews. Participants described feeling as though they should “just get on with it” (P05, P11). Some suggested that the perception of abdominal pain as a women’s issue contributed to the dismissal of their pain. Additionally, participants described doctors treating their pain as standard for their condition rather than listening to their individual pain experience or the ways in which pain impacted their lives.

“‘It shouldn’t be more than a mild discomfort, it shouldn’t really put you out.’ That was a little bit invalidating.” (P05)

“When it’s pelvic pain they’re just like, ‘oh, yeah, go home.’” (P08)

“You’re being really delicate, like a delicate little flower for not just putting up with the pain.” (P10)

The invisible nature of abdominal pain was discussed, with several participants relaying experiences of pain with a ‘visible’ cause being taken more seriously by their doctors. One participant (P07) described a comparatively positive experience of attending their hospital’s emergency department with a dislocated hip, versus the negative experience she had had in the same department when presenting with abdominal pain.

“Luckily, recently my hip dislocated, and everybody believed me, […] because it was a very visible, physical ailment. I was so shocked and almost gassing myself again, I’m like, ‘all these people seem to really believe you this time, maybe that means it’s not real.’ *(laughs)*” (P07)

Participants struggled to negotiate pain management solutions with their HCPs. These conversations typically centred on the type, effectiveness, and tolerability of medications for pain management. Some participants felt that the over-the-counter analgesics they were advised to use were ineffective for managing their pain. Others struggled to tolerate certain kinds of medications due to unique sensitivities or side-effects. Some participants were no longer able to tolerate certain medications they had used to manage their pain over many years. Experiences of contraceptive prescribing were mixed; some participants were told “to just go on the pill and that’ll sort everything,” (P08), while another participant was denied contraception for pain management. Participants with unspecified and/or gynaecological abdominal pain were particularly unsatisfied with their treatment.

“The pain killers they give you are nowhere near as strong as what you actually need.” (P13)

“I’m very sensitive to medication and I don’t agree with a lot of medication, and I had in the past the experience that they were not listening to [me about] certain pain killers or anti-inflammatories I can’t take.” (P12)

“I had to beg to be given anything.” (P06)

“In the same way I can’t ignore an email from a client, I don’t think they should be able to ignore my pain.” (P10)

Participants described varied emotional and psychological impacts experienced by participants following dismissive interactions with their HCPs. They discussed experiencing distress, confusion, hopelessness, embarrassment, and anger. Many described feeling “disheartened and saddened” (P14) by their initial negative experiences.

“I bawled my eyes out in the car afterwards.” (P14)

“I don’t think I’ve ever been as angry before in my life and I’ve never been as angry since. It was… It really, really upset me.” (P09)

Some participants discussed how they were particularly “disappointed” (P08) following dismissal by female doctors.

“I feel like that was a betrayal because she’s not just speaking as a doctor but also as a woman and saying, ‘oh, I also have a womb, […] that’s completely normal.’” (P10)

One participant discussed how the disruption her symptoms caused was amplified by how her initial doctor emphatically advised their normality, giving her “weapons to flay [her]self with” (P07).

“Very disruptive to my life and the fact like […] I thought everyone was just dealing with it better, because that first doctor came in so hard with the, ‘this is normal, this is the normal way to be.’” (P07)

Participants who had gone on to have more positive and validating experiences with HCPs emphasised how important this was for them, with “reassurance” (P01, P02) from doctors cited as something that dramatically improved their experience. Some participants discussed how they would prioritise seeing a doctor who made them feel heard over one with greater perceived medical expertise but who did not listen or was dismissive.

“Having a medical professional actually validating your experience is quite important. […] I don’t hate myself for having [premenstrual dysphoric disorder] now.” (P07)

“I wouldn’t necessarily trust their medical knowledge […] but I’d still trust them more than a lot of other doctors because I felt heard, and I felt cared for.” (P06)

### Theme 2 – “Bad Enough”? Costs of (Not) Seeking Help

Participants described the significant impact of abdominal pain on their daily lives. For most, pain was associated with adverse impacts on academic, social, and working life. Several participants reported that their symptoms interfered with even basic functions such as eating and sleeping.

“Yeah, I couldn’t really function. *(laugh)*” (P01)

Some participants spoke of how their symptoms and resulting hospital stays impacted their ability to attend lectures and required them to seek extensions for assignments while attending university. One discussed how, later, the same symptoms also interfered with her work.

“I had to [get] a doctor’s note because I wasn’t able to hand in an assignment on time. I could barely… I had no energy. […] So, it was awful, and I could barely get out of bed for a lecture. If I had a two-hour lecture, I’d make it to the second hour. Even if it was at twelve o’clock in the day, if it was a twelve to two lecture, I’d make it in for one o’clock. It was really, really not fun.” (P09)

“Multiple assignments had to get pushed back, and email multiple tutors to say I’m in hospital.” (P06)

“How am I supposed to be fully functioning in work if I’m constantly battling?” (P06)

Despite significant and wide-ranging impacts of pain on the lives of participants, many reported not wanting to go to their doctor until their symptoms were “bad enough” (P05). For many, this was due to the expectation that their concerns would be dismissed. For others, there was concern that there would not be any treatment options available to them. Those who had experienced medical trauma previously were particularly reluctant to seek care.

“To be like, ‘actually, this is bad enough for me to go to my GP and pay like 80 quid,’ I don’t want to be told that I’m due my period, y’know? If I’ve hit this point where I’m coming seeking medical attention for it, I know myself that it’s worse than a f***ing period.” (P05)

“[I wouldn’t go to the doctor] because knowing there’s nothing really they can do about it.” (P01)

“In the end they just gave me painkillers [that] I think I had at home. So, if it’s just that next time, y’know, for sure I might not go as soon.” (P04)

This idea that pain needed to be severe in order to warrant care was reinforced for some participants by their doctors. One participant described being denied a referral for specialist care because her symptoms did not meet a certain threshold of severity, despite the impact they were having on her wellbeing.

“I went into [the doctor] multiple times about hormonal issues, like skin, facial hair, bloating, all that kind of stuff, and it was put down to IBS and just bad periods with PCOS. She wouldn’t refer me to a gynae[cologist], she wouldn’t refer me to a dermatologist because I wasn’t bad enough.” (P08)

Each participant discussed the cost of healthcare and treatment as a barrier to seeking timely help and receiving appropriate care. Financial cost was identified by most as a reason to delay help-seeking. The cost of privately funded healthcare was discussed as prohibitive. Participants were also concerned about long waiting lists, as well as wasting their own time seeking help from providers who could not, or would not, help them to find relief from their pain. These personal costs contributed to increased dissatisfaction and anger with the healthcare system.

“I leave [the doctor] feeling like, ‘oh well, that was a waste of time.’” (P13)

“I don’t have the money to go in [to the doctor].” (P14)

“Yeah, it’s agitating because then, what do I do? Do I have to go privately to get that and pay for that or?” (P10)

“I was poor *(laughs)*, d’you know? I didn’t have the money to go private.” (P11)

Some participants indicated that they would gladly spend the time and money if they could access the kind of care that they felt they needed, but that no pathway to treatment was available.

“I’m happy to pay €70 for the next chat that we have. We say, ‘well, look, nothing came up in the ultrasound but let’s do bloods.’ I dunno what the next steps are. ‘Look, what we’re going to have to do is just wait for a while and see what happens.’ That would feel better than just… nothing.” (P10)

Despite delayed help-seeking being common in this sample, several participants talked about the importance of receiving an accurate diagnosis and appropriate timely treatment. Participants without a diagnosis to which they could attribute their abdominal pain, as well as those who had struggled to receive their diagnoses, spoke about the personal significance of this and empathised with others.

“So many people’s lives are kind of in the hanging because they’re like, ‘I don’t know what’s wrong with me.’ […] Some of them are actually in physical pain, like myself, and I… I would really like to get a diagnosis; I would really like to know what’s wrong with me.” (P13)

“I feel like there’s so many women suffering with [pain] on a day-to-day basis and they still, they’re not able to find a good doctor who can help them. They’re suffering for years, and they have no quality of life. If they had, like, a good doctor that could just sit down and really give them the time and listen to them, they could really find what’s wrong with them and help them and change their life.” (P03)

### Theme 3 – “Fight Your Case,” Fight For Care

This theme describes the experience of having to fight to be heard and to receive care from HCPs, as well as the ways in which participants accessed information and support from others to facilitate this fight. The perceived importance of self-advocacy when engaging with HCPs was evident throughout the interviews. Participants felt there is an onus of self-care to be borne by patients who are unable to rely on HCPs for care and support. Despite feeling that “there’s something wrong” (P02) and that their abdominal pain is “out of the ordinary” (P01), participants struggled to have their needs addressed by HCPs, who they felt did not take their concerns seriously.

“[Doctor said patient was stressed] It was like, yes, I’m stressed. Of course, I’m stressed, there’s sh*t going on. But then also, I have been stressed before. I know this is not my body’s immediate reaction to stress.” (P09)

“There’s something wrong, because, like, I have changed my diet and stuff. I have changed my diet and it’s still… it’s still happening no matter what I eat.” (P02)

“Respectfully, I have been having my period for over ten years now and I – this is not, like, this is not the normal pains.” (P10)

Social support facilitated participants in their fight for care, particularly support and encouragement from other women. Mothers assisted participants in advocating for themselves and were sometimes described as being the driving force behind their care. Many participants also received informational and emotional support from friends. Other women, including strangers, were described as playing pivotal roles in participants’ self-advocacy journeys.

“My Mam, my hero.” (P09)

“I would discuss […] with my friends, y’know, I’m always saying, ‘oh my, y’know, my stomach is sore,’ and they’re always saying, like, ‘go to get it sorted.’” (P02)

“Never met her before or nothing and she was so helpful and only for her I probably would still be in so much pain.” (P08, referring to an endometriosis advocate she heard on the radio and contacted for advice)

In contrast, some participants highlighted that societal perceptions of women’s pain acted as an impediment to them accessing care. For example, one participant described how the absence of discussion around menstruation when she was growing up impacted how she sought healthcare assistance for her pain.

“I found similarly to what I found at home, it was very much, ‘just get on with it, it’ll be grand.’ It’s like its period cramps essentially is what I – it was minimised to that, but it absolutely was not. So, that went on for years. […] Now, I did eventually find a really, really lovely healthcare practitioner, […] but I didn’t find her until I was almost in my late twenties.” (P11)

Social support provided some participants with the validation they needed to take the first step in seeking healthcare. Participants who had normalised their pain described how peers influenced their decision to seek help by affirming that their pain was debilitating. Having someone else confirm that their pain was severe and warranted investigation gave impetus to help-seeking.

“I was like, ‘it’s mild discomfort, that’s all this is,’ so it wasn’t until someone was like, ‘that was actually scary to see, you were holding your breath, couldn’t stand up. You were, like, crawling to the bathroom… it was pretty bad, you should go to A&E. That was really bad.’” (P05)

While informational social support was helpful, participants discussed feeling the need to do additional self-directed research on their symptoms. The main purposes of information-seeking were described as “wanting to back yourself up” (P13), to self-diagnose, and to manage symptoms, especially when support from HCPs was lacking. For most, information-seeking was motivated by a lack of trust in HCPs.

“I did do my research before I went in, to kind of… be, not defensive, but to be… to prove… it is defensive, I guess. […] I didn’t know if they would take my pain seriously otherwise.” (P13)

“I can’t trust a doctor to diagnose me.” (P06)

“It doesn’t seem like [HCPs] actually know what they’re talking about at all.” (P10)

Similarly, participants reported having to request referrals to certain specialists or for specific diagnostic tests to receive care. An unprompted referral from a GP occurred for only one participant in this sample. This confirmed many participants’ beliefs that the responsibility for their care is on them, and that, if they are not armed with detailed information about their symptoms and the healthcare system, they will not receive appropriate care.

“My Mam asked could I get a referral, because he [HCP] thought it was just period pain, but we knew it was more.” (P03)

### Theme 4 – “Out of the Loop” – Systemic Barriers to Care

Many participants discussed structural issues within the Irish healthcare system as barriers to receiving effective care. There was a particular focus on issues of communication. Participants discussed how communication within and between healthcare departments and services was lacking, with one participant commenting that, when she was admitted to the emergency department for abdominal pain, “at no point in time had they gotten on to gynaecology to take a look, despite the fact that I attend there for gynaecology,” (P06). Some participants expressed desire for a more “community based,” (P12), “collaborative,” (P11), and “holistic” (P12) healthcare approach, which they believed might improve the situation for both patients and HCPs.

“I don’t even know if [my GP has] been told about the endometriosis. I’m very out of the loop.” (P05)

“A lot of people are not getting the treatment they need, and they deserve, because the communication just isn’t there.” (P09)

“We’ll figure this [systemic communication issue] out and that will reduce the expectations of the patients, and as well for the doctor to understand absolutely everything. […] But also, I think it removes some of the stress that healthcare practitioners may be under.” (P11)

Administrative issues were frequently discussed, with issues around appointment notifications being highlighted as particularly problematic. There was a sense of frustration from participants who felt penalised by the lack of clear communication from the health service and avoidable administrative errors.

“I wasn’t sent a reminder or anything, which I think is a little bit off, but I suppose it was my own fault for missing the appointment. […] To then get a letter like, ‘eh, you missed your appointment, well go f*** yourself, we’re not seeing you anymore.’” (P05)

“They actually told me, ‘you’re not eligible anymore because you missed the appointment.’ Even though I tried to explain to them […] ‘you didn’t send [the letter] to my correct address, and you never emailed me or called me.’” (P14)

Despite their frustrations with the healthcare system, participants displayed empathy for the HCPs. They felt it was important to recognise that HCPs “are human beings at the end of the day,” (P11) and their jobs are difficult and stressful. Participants acknowledged that the systemic failures in the Irish healthcare system place a significant burden on HCPs and impact their ability to provide adequate care. Some participants described feeling ‘guilty’ (P10) when relaying negative healthcare interactions, in part due to their understanding that the barriers to accessing care are bigger than any individual, but also an internalised sense that their pain may not be worthy of care within a system under strain.

## Discussion

The current study aimed to explore the help-seeking experiences of women with abdominal pain in Ireland. Participants’ experiences were largely negative, characterised by feelings of dismissal and invalidation, struggle to have symptoms taken seriously, and frustration with systemic barriers to diagnosis and treatment. Internalised normalisation of pain was apparent in this sample, with many participants describing a perceived need for their pain to reach a certain (high) threshold of severity, as evaluated by themselves or others, before seeking medical attention. Participants felt that they were primarily responsible for making sure they received the care that they needed. Despite feeling frustrated with their healthcare experiences, participants acknowledged that many of the barriers they faced were systemic and expressed empathy for HCPs operating within a flawed system.

Past experiences of perceived dismissal by HCPs affected participants’ willingness to seek healthcare. Participants emphasised the importance of feeling heard by HCPs, with some stating that their doctor’s ability to make them feel heard was more important to them then their medical expertise. This is consistent with previous literature, which highlights that listening is essential for building a trusting patient-doctor relationship that facilitates open communication [22, 42]. Resource limitations within the healthcare system, particularly in public healthcare, place extreme pressures on HCPs’ time, which may exacerbate patients’ feelings of dismissal. Participants suggested that a more collaborative approach to healthcare could be beneficial not only for their own health, but also for HCPs. This could alleviate some of the pressures faced by HCPs, and GPs in particular, to be “expert in everything.”

All women in this study emphasised the importance of self-advocating in order to be taken seriously by HCPs. Although women have been shown to have greater self-advocacy intentions, these do not appear to be associated with less negative clinical experiences regarding pain [24], suggesting that disparities in care cannot be attributed to women failing to self-advocate. It is likely that these disparities are in part due to the normalisation of women’s abdominal pain. Both women and doctors may assume that their abdominal pain is normal, even when that pain is severe [17, 20, 43]. This is also a systemic issue, whereby pressures on the healthcare system result in the de-prioritisation of “non-urgent” or “non-malignant” issues, without due consideration for the impact on functioning or quality of life. Clearer conceptualisation of health and illness according to the International Classification of Functioning, Disability and Health (ICF) [44] in the healthcare system, whereby activity limitations and participation restrictions are central, could help alleviate some of these issues.

### Implications of the findings

The current findings have important implications for future research. This qualitative study provides important insight into the lived experience of women seeking care for abdominal pain in Ireland. Large-scale quantitative research is now needed to better understand the extent of the issues identified in the current study. Probability sampling approaches that account for key demographic factors like age, socioeconomic status, and geographic location should be used to ensure findings are as robust as possible. This research should also consider the experiences of male patients presenting with abdominal pain to better understand the gendered and non-gendered issues in this context. Quantifying the extent of gender-based disparities in assessment and treatment of abdominal pain is necessary to underscore the importance of future investment in efforts to make healthcare in Ireland more equitable. Future research should also aim to understand the views and perspectives of HCPs on how care is delivered to women with abdominal pain in Ireland. The experiences of HCPs are typically overlooked in this literature, which may result in a biased assessment of the barriers and facilitators of care for women with pain. Understanding their perspectives as well as those of patients would enable the development of a more comprehensive approach to addressing these issues.

Our findings also have implications for healthcare practice and public health. Participants in this study had a wide variety of underlying causes for their abdominal pain, and as such encountered several different medical specialists. It is therefore particularly striking that participants’ experiences of engaging with the healthcare system were so similar. This demonstrates that the issues raised in this study are not specific to any one medical discipline, but rather reflective of the healthcare system as a whole. Medical education and professional development opportunities are needed to ensure HCPs are equipped to understand and treat women’s abdominal pain, and to refer to the appropriate specialists as needed. Consideration of the impact of pain on women’s functioning is essential in this regard; therefore, these initiatives should be informed by the ICF [44] and emphasise the impact of unmanaged pain on quality of life. Furthermore, it was apparent in this study that participants internalised normative views of women’s pain, which affected their help-seeking behaviours. Public health campaigns to promote appropriate help-seeking for pain that interferes with daily functioning should be developed to ensure women with abdominal pain receive pain management support and timely diagnosis of any underlying pathology.

### Strengths and limitations

Certain limitations should be considered when interpreting the findings of this study. Participants self-selected into the study, which means the findings may be subject to certain biases. In particular, individuals with negative healthcare experiences may have been more motivated to take part. Participants were also white, well-educated, and largely had the means to access private healthcare. Therefore, the research lacks insight into potential cultural or sociodemographic factors that may affect help-seeking experiences. That said, cost was discussed by most participants as a prohibitive factor. Ireland has a two-tiered public-private health service [26, 27]. Primary healthcare costs are payable by roughly 60% of the population, at an average cost of approximately €50 per GP visit [45], with over one-third of private patients paying up to €75 per visit [46]. Though the General Medical Services Scheme exists to allay some of these costs for individuals on a reduced income [47], it is likely that those who are less well-off financially may experience additional barriers to accessing care [48]. Additionally, most participants were between 18 and 29 years of age, which may limit the generalisability of findings to other age groups. Older women may have different healthcare needs, expectations, and experiences, and engage in different healthcare-seeking behaviours compared to younger women. That said, the current findings provide important insight into the experiences of young women, who may face unique barriers to receiving adequate care [49]. Finally, only cisgender women took part in the study. Experiences of and barriers to healthcare for LGBTQIA + individuals with female anatomy who experience abdominal pain are likely to be different from those of cisgender women [50, 51]. Future research should aim to understand the experiences of diverse groups of individuals to make the research literature and healthcare practice more inclusive.

Limitations notwithstanding, the current study makes an important contribution to the literature on help-seeking experiences for women with abdominal pain. Steps were taken to ensure the analysis was conducted rigorously and that principles of reflexivity were adhered to throughout the research process, which strengthens the validity of our conclusions. Inclusion of women with different kinds of abdominal pain, including unspecified pain, strengthens the research. Condition-specific approaches run the risk of forcing conclusions that challenges to accessing healthcare may be discipline-specific. While certain specialties or treatments may be less accessible due to, for example, resource limitations, our findings provide evidence that gender-specific challenges to receiving care for abdominal pain are prevalent across the healthcare system. This strengthens our assertion that system-wide change is needed to promote the health and wellbeing of women experiencing pain.

## Conclusions

Participants described mostly negative experiences of seeking healthcare for abdominal pain. Previous experience of dismissal of symptoms and social perceptions influenced participants’ willingness to engage with healthcare services. Women may internalise the idea that severe pain is normal and attempt to tolerate it without effective support, potentially to their long-term detriment. Participants’ experiences with healthcare reinforced their view that self-advocacy is essential to allow them the chance to receive care for their pain. There are systemic issues at play within the Irish healthcare system that limit women’s ability to access abdominal pain management support. Public health campaigns that challenge normative views of women’s abdominal pain as not warranting healthcare intervention and promote appropriate help-seeking for disabling pain are needed. Education and training for HCPs on the Gender Pain Gap and its implications for patient care, as well as clear referral pathways for women presenting with abdominal pain, are needed to ensure more equitable healthcare delivery for individuals with pain in Ireland.

### Electronic supplementary material

Below is the link to the electronic supplementary material.

Supplementary Material 1

## Data Availability

Data supporting the present findings are not publicly accessible due to ethical responsibilities for data protection. Pseudonymised data, however, may be available on reasonable request to the corresponding author.

## Abbreviations

A&E: Accident and Emergency Department
GDPR: General Data Protection Regulation
GP: General Practitioner
HCPs: Healthcare Professionals
IBD: Inflammatory Bowel Disease
IBS: Irritable Bowel Syndrome
ICF: International Classification of Functioning, Disability and Health
LGBTQIA+: Lesbian, gay, bisexual, transgender, queer, questioning, intersex, or asexual
PCOS: Polycystic Ovary Syndrome
PMDD: Premenstrual Dysphoric Disorder
