# Supplementary material for: Women’s experiences of seeking healthcare for abdominal pain in Ireland: a qualitative study
Source: BMC Womens Health. 2024 Mar 7;24:166. doi: 10.1186/s12905-024-02995-3 (PMC10921746; doi:10.1186/s12905-024-02995-3)
Supplement: Supplementary file 1 — Supplementary Material 1 [file 12905_2024_2995_MOESM1_ESM.docx]

**Additional File 1**

**Interview Guide**

**Introduction**

*Welcome the participant and thank them for participating in the study. Introduce yourself and the topic of the research. Reassure them about their anonymity, highlight that they can pause or end the interview at any time, and emphasise that they do not have to answer any questions they do not wish to. Ask the participant if they have any questions. Get affirmative consent to take part.*

**Build Rapport**

- To start off can you tell me a little bit about yourself.
- Name.
- Age.

**Questions/Topics**

- Have you experienced pain in your abdomen? / Have you experienced abdominal pain?
- Have you ever gone to your GP specifically to discuss this pain? Or another healthcare practitioner? / Have you brought this pain up with your GP or another healthcare practitioner?
- Can you tell me a little bit about your experiences of visiting your GP?
- Do you feel/think they know you well?
- Do you feel there are some things you cannot / should not discuss with / mention to your GP?
- How do you think your doctor thinks about your pain?
- What are your expectations when dealing with healthcare professionals?
- How do you feel about going to the doctors/visiting your GP/a healthcare professional?
- How do you prepare for interactions with medical personnel/your GP?
- Do you expect to be listening?
- Could you describe how you feel about visiting the doctor/appointments with healthcare professionals?

**Suggested Follow-up/Probes**

- Could you tell me more about that feeling?
- What makes you say that?
- Could I ask you to expand on that a bit more?
- Why? / May I ask you why?

**End of Interview**

*Thank the participant for their time. Remind the participant that they are welcome to contact the interviewer if they have any questions or concerns.*
